# An Acquisition Scheme Based on a Matched Filter for Novel Communication and Navigation Fusion Signals

**DOI:** 10.3390/s17081766

**Published:** 2017-08-02

**Authors:** Zhongliang Deng, Jun Mo, Buyun Jia, Xinmei Bian

**Affiliations:** School of Electronic Engineering, Beijing University of Posts and Telecommunications, No. 10 Xitucheng Road, Haidian District, Beijing 100876, China; dengzhl@bupt.edu.cn (Z.D.); jiabuyun@bupt.edu.cn (B.J.); bianxinmei@bupt.edu.cn (X.B.)

**Keywords:** communication and navigation fusion signal, PRN sequence, TPRN sequence, matched filter algorithm

## Abstract

In order to enhance the positioning capability of terrestrial networks, a novel communication and navigation fusion signal is proposed. The novel signal multiplexes the communication and navigation signal in the same frequency band, and the navigation system is superimposed on the original communication system. However, the application of pseudorandom noise (PRN) sequences in the navigation system is limited by the communication clock period. Taking the application of PRN sequences limited by the clock period as objects, the present study analyzes truncated PRN (TPRN) sequences. PRN sequences with a TPRN sequence as the navigation signal can overcome the communication system clock period limitation. Then, a matched filter algorithm with double detection (MFADD) is proposed to acquire the novel signal. The matched filter method is applied to the proposed algorithm to determine the start code phase of TPRN. Monte Carlo simulations and real data tests demonstrate the effectiveness of the proposed algorithm for the designed signal.

## 1. Introduction

Global navigation satellite systems (GNSS) can provide high accuracy positioning information in an open outdoor environment, but fail in urban canyons and indoor environments [1,2]. Terrestrial networks have wide outdoor and indoor signal coverage in the city, and it can be considered as a way of compensating for the shortcomings of GNSS [3]. As a typical terrestrial network 2G/3G/4G mobile communication systems can provide indoor positioning information, but the positioning accuracy is too poor to meet the needs of most location-based services (LBS) [4,5]. A novel communication and navigation fusion signal could solve the problem by multiplexing the communication and navigation signal in the same frequency band. The communication signal and the navigation signal are then transmitted at the same time using the same frequency band, and the existing communication system only needs to be simply modified.

**Figure 1:**
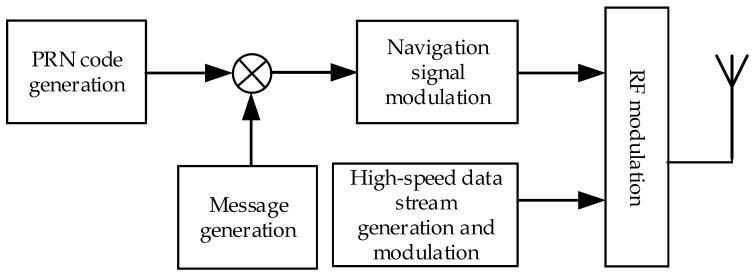
Flowchart of communication and navigation fusion signal generation.

This paper mainly studies a multimedia broadcasting system named China mobile Multimedia Broadcasting (CMMB), and modifies it to achieve positioning functionality. At the beginning of customization, the CMMB standard did not take full account of using this signal for positioning. The CMMB ground coverage network adopts a single transmitter station and signal frequency network structure, and the reception sensitivity of the CMMB receiver is −95 dBm [6]. If the receiver cannot receive three or more navigation signals from the base stations, it cannot be located. In addition, the basic information of the base stations for positioning (such as station location, height, delay correction, etc.) is not designed in the CMMB standard. In general radio pseudorange measurement, spread spectrum signals are used in the ranging signal, whose spread spectrum gain is generally higher than that of the multimedia broadcasting signal. Then the ranging signal has more effective coverage than the broadcasting signal. Therefore, in this proposal the navigation system using spread spectrum signals and the communication system using CMMB are multiplexed in the same frequency band. The navigation system is a direct-sequence spread spectrum code division multiple access (DSSS-CDMA) system employing binary phase shift keying (BPSK) modulation, and the basic information of the base stations for positioning is added in the navigation system, and the communication system maintains the original state without modification. Figure 1 shows the flowchart of the communication and navigation fusion signal generation. In order to achieve high accuracy positioning, the reception sensitivity of the receiver must be improved, and then three or more navigation signals can be demodulated even if only one broadcast signal is demodulated. 

In a DSSS-CDMA system, the spectrum spread rate is held in the clock period, and the sequence length of the address code relies on the quotient between clock rate and information rate [7]. Since the communication system is not modified, the navigation system needs to share the same frequency clock with the communication system. Although the phase locked loop (PLL) can generate the desired output frequency by frequency-doubling or frequency-dividing, output frequency without deviation is only for partial incoming frequency, so the application of pseudorandom noise (PRN) sequences is limited in navigation system. Reference [7] gives a solution to satisfy the particular requirements of navigation systems for using PRN sequences, namely the solution of truncating segments of PRN sequences to generate truncated PRN (denoted as TPRN) sequences. However, the nearly-optimal correlation properties of PRN sequences are strictly dependent on their exact length, so truncation causes the appearance of a truncation noise in their autocorrelation function [8,9]. Therefore, TPRN sequences destroy the anti-interference and anti-jamming capability of the navigation system. Although extending the integration time can compensate for the capability of the system to resist anti-Gaussian white noise (GWN) interference, it has no effect on anti-multiple access interference (anti-MAI). Moreover, the algorithms which generate TPRN sequences with minimum truncation noise are complex. This paper designs a novel signal that multiplexes communication signals and PRN sequences with TPRN sequences to overcome the above problems.

The acquisition process is the first stage of the digital part in positioning receivers, intending to acquire received navigation signals and get a coarse estimation of residual carrier frequency and code delays [10]. In DSSS-CDMA system, the usual parallel acquisition methods are: matched filter method [11,12,13] and cyclic correlation method [14,15,16]. With the matched filter method, the received signal is correlated with a locally generated signal of the modified digital broadcasting base station (MDBBS) to perform acquisition. If the cross-correlation peak value crosses the predefined threshold, the acquisition process is successfully completed. In addition to the above condition, it is necessary to determine the TPRN sequence phase for the designed navigation system. If the TPRN phase cannot be determined, the tracking code loop will be out of lock and the received signal cannot be stably tracked for a long time. Conversely, if the TPRN phase is detected, we can use PRN and TPRN for long coherent integration to improve the tracking sensitivity in the tracking process. The cyclic correlation method based on fast Fourier transform (FFT) in [14] can quickly calculate the correlation, but the TPRN sequence will cause a significant degradation on the autocorrelation peak value, which is a phenomenon known as spectral leakage [17]. Coherent, non-coherent and differentially coherent integrations are performed to obtain the maximum signal-to-noise ratio (SNR) [18,19]. However, the need to distinguish the TPRN sequence phase and bit transitions limits the length of the signal record to be coherently integrated. In this paper, a novel matched filter algorithm with double detection (MFADD) is proposed for the designed signal acquisition. MFADD algorithm extends effective non-coherent integration time to find the possible start code phase of TPRN, and then performs successive comparisons of the coherent integration values with the predefined threshold to determine the starting code phase of TPRN. Monte Carlo simulations and real data tests are performed to verify the effectiveness of the proposed algorithm.

The following of this paper is organized as follows: in Section 2, the fusion signal model is given and acquisition method is analyzed. Then, non-coherent integration extending method and successive comparisons method are presented in Section 3. Based on these two methods, the MFADD algorithm is proposed. Section 4 gives simulation results, real data tests and performance analysis of the proposed MFADD algorithm. Finally, Section 5 concludes the work.

## 2. Signal Model and Acquisition Method

In this section, a novel communication and navigation fusion signal is designed which multiplexes communication signals and PRN sequences with TPRN sequences. The acquisition method of the designed signal is described and analyzed.

### 2.1. Comnunication and Navigation Fusion Signal

**Figure 2:**
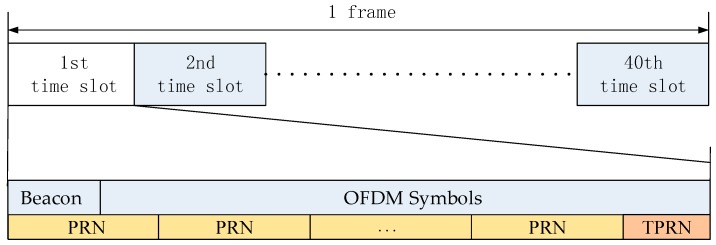
Structure of the communication and navigation fusion signal.

The communication and navigation signal is multiplexed in the same frequency band. Figure 2 shows a structure of the communication and navigation fusion signal. The communication system is CMMB, and CMMB transmits the broadcasting signal using orthogonal frequency division multiplexing (OFDM) modulation [20]. The CMMB signal is one frame per second, divided into 40 time slots, each with a 25 ms of length, including one beacon and 53 OFDM symbols. The beacon contains the TxID transmitter identification signal and two identical synchronization signals. Since the CMMB uses single transmitter station or signal frequency network structure, synchronous signals and data symbols transmitted by all base stations are the same. TxID identifies the number for the base station. When the TxID is odd, it indicates the area code. When the TxID is even, the number of the base station in the area is indicated. However, the TxID in the existing CMMB has not been put into use, and the receiver is not concerned with the TxID. In this structure, the multiplexed navigation signal is superimposed on the digital broadcasting signal. Considering the transmission of the fusion signal and the time of demodulating the navigation message, an equal length spread code navigation signal is superimposed on each time slot structure of the broadcasting signal, and the navigation signal energy is lower than the broadcasting signal by 20 dB. The length of one-bit data is the same as the length of one-time slot digital broadcasting signal, and the navigation signal time slot is perfectly aligned with the communication signal. These two systems share the same 1 pps pulse signal to adjust the signal broadcasting, and then it achieves the synchronization between MDBBSs. The navigation signal of one-time slot consists a number of repetitive PRN sequence and a TPRN sequence. The PRN and TPRN sequence belong to the same family, and the TPRN sequence is a segment of other PRN sequence where some bits have been truncated, and the TPRN sequence is an arbitrary continuous segment of the complete PRN sequence. 

The novel signal of the *n*th time slot can be expressed as:

(1) $$s_{n}^{\left(i\right)}(t)=\left\{ \begin{array}{lll} s_{CMMB}(t)+c_{PRN}^{\left(i\right)}(t) & & (n-1)T_{F}\leq t<nT_{F}-T_{TPRN}\\ s_{CMMB}(t)+c_{TPRN}^{\left(i\right)}(t) & & nT_{F}-T_{TPRN}\leq t\leq nT_{F}\\ 0 & & \;others \end{array}\right.,$$

where, superscript *i* stands for the MDBBS number, $s_{CMMB}$ is the CMMB signal, $c_{PRN}(\cdot )$ and $c_{TPRN}(\cdot )$ denote two Gold codes, $T_{F}$ is the time length of the time slot, $T_{TPRN}$ is the time length of the TPRN sequence.

The novel signal of *i*- MDBBS is:

(2) $$s^{\left(i\right)}(t)=\sum_{n=-\infty }^{\infty }d_{n}^{\left(i\right)}(t)s_{n}^{\left(i\right)}(t),$$

where, $d_{n}(t)$ denotes the navigation data sequence. The RF signal sent by *i*-MDBBS is:

(3) $$S^{\left(i\right)}(t)=s^{\left(i\right)}(t)\cos(2\pi f_{c}t+\varphi_{0,i}(t)),$$

where $f_{c}$ is the carrier frequency, $\varphi_{0,i}(\cdot )$ is the initial phase.

### 2.2. Acquisition Method

Since the TxID is not used, the receiver cannot obtain the MDBBS information by demodulating OFDM. At the same time, the receiver cannot simultaneously demodulate the OFDM signals transmitted by three or more MDBBSs, so we can only use the added navigation system to achieve the CMMB positioning. Thanks to PRN sequence orthogonality, incoming signal of all received MDBBSs can be analyzed separately [21]. The digital broadcasting signal is received by RF antenna, and the output of RF antenna is written as:

(4) $$r(t)=\sum_{i=1}^{N}A_{i}s^{\left(i\right)}(t-\tau_{i})\cos(2\pi (f_{c}+f_{d,i})t+\varphi_{0,i}(t))+\omega (t),$$

where, *N* is the sum of the received signals that are sent by *N* different MDBBSs, *A_i_* is the signal amplitude, *τ_i_* is the incoming code delay, *f_d,i_* is the incoming Doppler shift, w(t) stands for the additive Gaussian white noise (AWGN) component with zero mean (*μ* = 0) and variance ($\sigma_{n}^{2}$).

After the digital broadcasting signal reaches the receiving antenna, the received signal is converted to intermediate frequency (IF) through the amplifier, mixer and filter, and finally IF signal is output to baseband signal processor by analog-to-digital converter (ADC) model. The communication signal is filtered out. Neglecting the quantization effect, the incoming signal of baseband signal processor can be expressed as:

(5) $$r(nT_{s})=\sum_{i=1}^{N}A_{i}^{\prime }c_{Nav}^{\left(i\right)}(nT_{s}-\tau_{i})e^{j2\pi (f_{IF}+f_{d,i})nT_{s}+\varphi_{0,i}}+\omega (n),$$

where, $A_{i}^{\prime }$ is the signal amplitude after ADC, $T_{s}$ is sampling time, $f_{IF}$ denotes the intermediate frequency, $c_{Nav}(\cdot )$ is the navigation signal and $r(n)=r(nT_{s})$.

**Figure 3:**
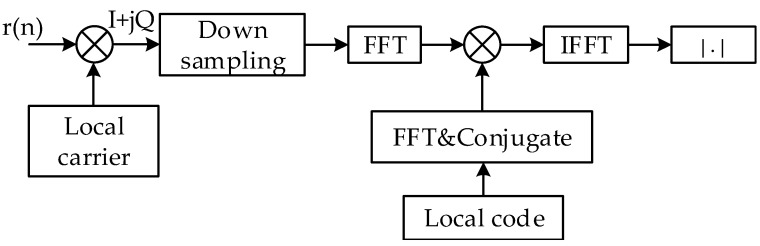
Flowchart of cyclic correlation method.

Acquisition process is the first stage of the digital part in the positioning receiver, which is to get initial estimate of the residual carrier frequency and code delay of all the received MDBBSs. Generally, acquisition techniques in the code domain used inside the receivers are based on the matched filter method or the cyclic correlation method. For the sake of convenience, it is assumed that the carrier has been determined and does not have any effect on the code domain searching. The cyclic correlation method is to realize the parallel acquisition of the code domain by using the cyclic correlation theorem in digital signal processing. The cyclic correlation results of two periodic sequences can be obtained by FFT and IFFT operation [15]. Figure 3 depicts the structure of the cyclic correlation method. This method performs the FFT operation on the down-sampled in-phase (I) and quadrature (Q) signals and multiplies the FFT result of the local complex code, and then performs the IFFT operation to finally obtain the correlation value on all the code phases. The difference with the matched filter method is that the cyclic correlation method uses FFT to operate in the frequency domain.

**Figure 4:**
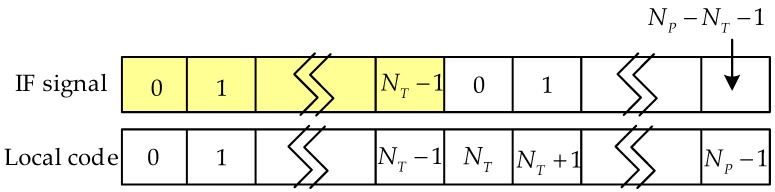
The TPRN leads to the nature of the correlation attenuation.

**Figure 5:**
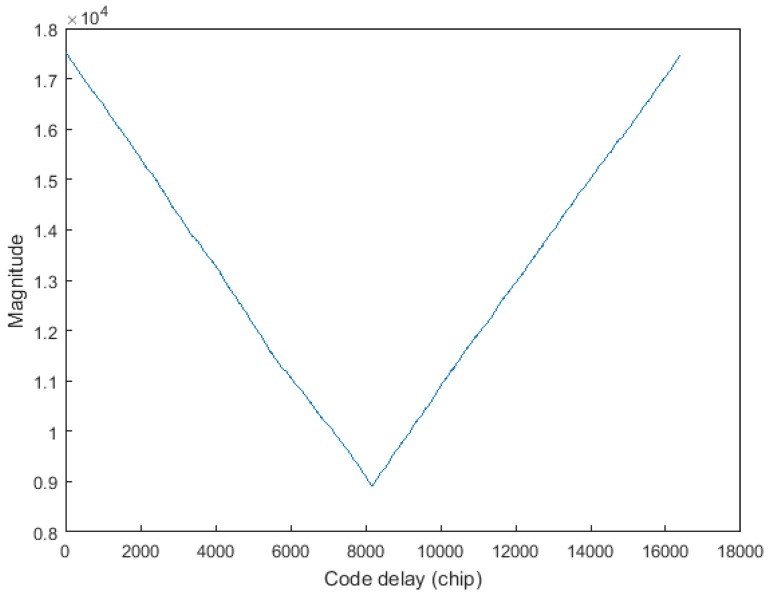
Zero-padding leads to the autocorrelation attenuation.

**Figure 6:**
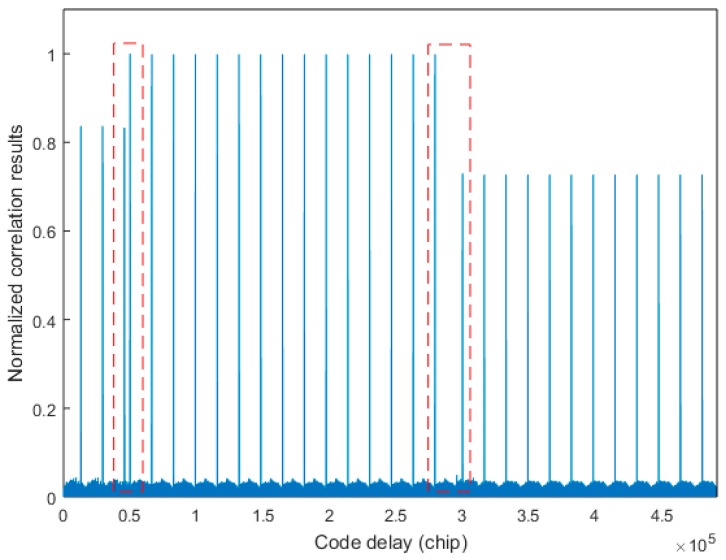
Correlation using the cyclic correlation method.

Since the exact code phase of the received signal is unknown, the TPRN position of the local signal and the received signal are not the same, resulting in the destruction of the cyclicity between the pseudocode of the received signal and the local replica code. Thereby, the correlations between the received code and the local replica code are attenuated, resulting in distortion of the autocorrelation peak shape. Figure 4 shows the nature of the autocorrelation attenuation due to the addition of the TPRN signal, where the yellow part of the figure is the TPRN sequence and the rest is PRN. In Figure 4, $N_{T}$ is the length after the TPRN sampling and $N_{P}$ is the length after the PRN sampling. The actual use of the FFT is an integer power of two, the zero-padding method and the up-sampling method also have the above-mentioned problems. Figure 5 depicts the peak energy attenuation corresponding to each phase in the case of the end zero-padding, which uses Gold code with 8191 length and 2 time sampling. When the offset chip is 8191 semi-chip, the energy attenuation is about 6 dB. Figure 6 shows the autocorrelation peaks attenuation because of two reasons above. The simulation conditions of Figure 6 are: the designed signal consists of 15 full cycle PRN sequences and one TPRN sequence, PRN of the designed signal and the local replica code are the same, w(n)=0, $f_{d}=f_{IF}=0$, the length of PRN sequence is 8191 and the sampling rate is two times. The autocorrelation peak spacing changes in the red dashed box in Figure 6 are due to the presence of the TPRN sequence, but they are not regular. Therefore, if we use FFT for the designed signal acquisition, we must consider other complex algorithms to solve this problems. 

**Figure 7:**
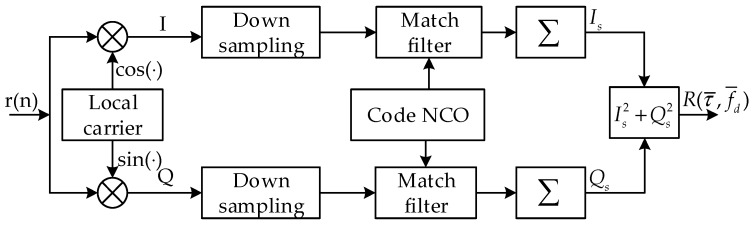
Flowchart of the matched filter method.

Another common parallel code acquisition technique is the matched filter method. This method is where the local signal generator generates local replica codes with all different code phases in a PRN period, and each output value corresponds to the correlation result between local replica codes and the received signal, as shown in Figure 7. Usually, the local replica codes are fixed, and the received signal is correlated with the local codes in sequence. It is possible to perform the correlation operation of the received signal and the local signal with all possible code phases in one PRN period. The final output result is expressed as the complex-valued circular correlation between the incoming signal and the local generated signal, and shown as:

(6) $$R(\bar{\tau },{\bar{f}}_{d})=\frac{1}{T_{c}}\sum_{n=0}^{T_{c}-1}r(n)c(n-\bar{\tau })e^{-j2\pi (f_{IF}+{\bar{f}}_{d})nT_{s}},$$

where $T_{c}$ is the coherent integration time, which is the same as the PRN sequence period; $c(n-\bar{\tau })$ is the local replica of PRN sequence; $\bar{\tau }$ and ${\bar{f}}_{d}$ are the tentative code delay and the residual carrier frequency respectively. 

**Figure 8:**
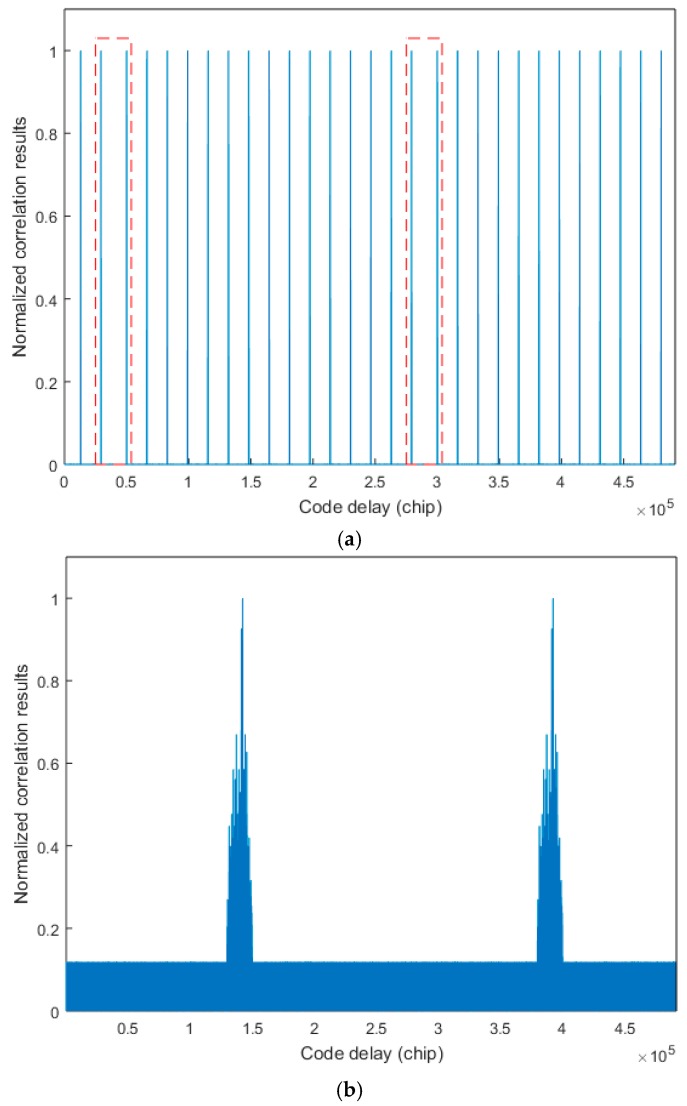
Correlation using the matched filter method: (**a**) autocorrelation results and (**b**) cross-correlation results.

The simulation conditions in Figure 6 and Figure 8 are the same, but the autocorrelation peak values in Figure 8a are not attenuated. The spacing between two autocorrelation peaks in the red dashed box in Figure 8a is larger due to the presence of the TPRN sequence, and they are regular. Therefore, we can use this rule to find the exact location of the TPRN in the case of hardware resources allowed. In addition, Figure 8b shows the cross-correlation values. As the TPRN signal is added, the balance of the original PRN sequence is destroyed, so there are many irregular peaks in the cross-correlation values, but the appearance of these peaks does not affect the use of the above rule on the TPRN phase search. In the traditional matched filter method, the maximum correlation of this method is compared to a predefined threshold to make a decision regarding the presence or absence of the searched MDBBS. However, it does not determine the specific location of the TPRN, so we propose a novel algorithm to determine the existence of the searched MDBBS and the specific location of the corresponding TPRN. 

## 3. Matched Filter Acquisition Algorithm with Double Detection (MFADD)

As discussed in the previous section, the presence of the searched MDBBS can be found using a matched filter method, but the TPRN sequence phase cannot be detected. In this section, a novel algorithm named MFADD is proposed to solve the above problem. In order to focus on the TPRN sequence phase detection problem, residual carrier alignment is presumed in the following parts of this section. Considering the limited receiver hardware resources and the complexity of the algorithm, we adopt two steps to acquire the designed signal: the first step is to determine the presence of the searched MDBBS or not, and give reference code phase of the corresponding TPRN to the second step; the second step is to determine the specific location of the TPRN of the existing MDBBS.

### 3.1. Reference Code Phase of the Presence MDBBS

The first step of the MFADD is to detect the existence of the searched MDBBS or not. Suppose that a time slot includes *K* periods of full PRN sequence and a TPRN sequence, the PRN sequence period has $N_{PRN}$ chips and the TPRN sequence has $N_{TPRN}$ chips. In order to increase the calculation speed and avoid the problems of using FFT, the correlation is evaluated by parallel correlators. The output of the parallel correlators are:

(7) $$Corr(m)=\sum_{n=0}^{N_{PRN}-1}r(n+m)c(n),$$

where $m=0,1,\cdots ,N_{PRN}-1$, $f_{IF}=0$ and $Corr(m)=R(\bar{\tau },0)$.

**Figure 9:**
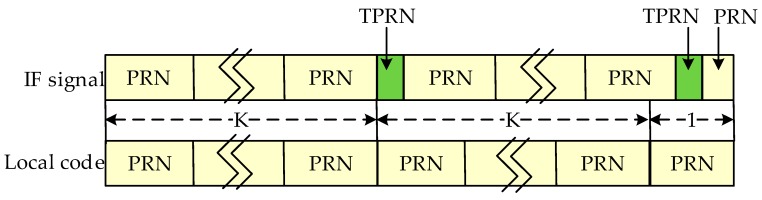
A condition of MFADD.

We can detect the presence of the searched MDBBS by comparing the maximum correlation value with the set threshold, and the distribution of correlations is similar to that shown in Figure 8. In the case of the TPRN sequence, the processing time is more than a slot time at least. When coherent integration is performed, data bit sign transition problem and small frequency search interval will appear. Therefore, non-coherent integration is performed to calculate the reference code phase. Considering the impact of noise on acquisition operation and the operation includes at least one full TPRN, we set 2*K* + 1 PRN sequence periods as the non-coherent integration time to determine the reference code phase. Figure 9 shows a case where the received signal is in parallel correlation with the local replica code, but the local replica code only needs a full PRN sequence in the practical application. After performing coherent correlation on each $N_{PRN}$ chips, a correlation vector is obtained and expressed as:

(8) $$\overset{⟶}{Corr(\bar{\tau })}=[Corr_{1},\ldots ,Corr_{k},\ldots ,Corr_{2K+1}],$$

where $Corr_{k}$ denotes the correlation results of *k*th integration period from the parallel correlation acquisition method by Equation (7). Thus results of the 2*K* + 1 non-coherent integration times can be expressed as:

(9) $$S(\bar{\tau })=\sum_{k=1}^{2K+1}{\left|Corr_{k}\right|}^{2},$$

where k=1,2,⋯,2K+1. S(⋅) denotes the non-coherent accumulation envelop. When the PRN of the received signal and the local replica code are the same, there is an obvious autocorrelation peak in each PRN period and the autocorrelation peak spacing in the same time slot is same. There is only one obvious autocorrelation peak in the same time slot using non-correlation operation, but there are two obvious autocorrelation peaks in two time slots using non-correlation operation due to the presence of TPRN. 2*K* + 1 PRN sequence periods consist of three time slots, so the envelop of S(⋅) appears three significant peaks with certain laws. Otherwise there will be no such phenomenon, and then we reacquire using the other PRN as the local replication codes. The phases of these three peaks can meet one of the following conditions:

(10) $$\{\begin{array}{c} P_{2}-P_{1}=N_{TPRN}\\ P_{3}-P_{2}=N_{TPRN} \end{array},$$

(11) $$\{\begin{array}{c} P_{2}-P_{1}=N_{TPRN}\\ P_{3}-P_{1}=N_{PRN}-N_{TPRN} \end{array},$$

(12) $$\{\begin{array}{c} P_{3}-P_{2}=N_{TPRN}\\ P_{3}-P_{1}=N_{PRN}-N_{TPRN} \end{array},$$

where $P_{1},P_{2},P_{3}$ are the phases of the three autocorrelation peaks arranged in the order in which they appear. $P_{1},P_{2}$ and $P_{3}$ also indicate that they are aligned with the local code at this time or before. Taking into account the convenience of counting, $P_{3}$ is selected as the reference code phase $P_{r0}$. However, in the real environment, envelop of non-correlation results will may appear two significant peaks due to GWN and the incoming signal phase. These two phases of the peaks will meet any of the following two conditions:

(13) $$P_{2}-P_{1}=N_{TPRN},$$

(14) $$P_{2}-P_{1}=N_{PRN}-N_{TPRN},$$

where $P_{1}$ and $P_{2}$ are the phases of these two peaks arranged in the order in which they appear. Then $P_{2}$ is selected as the reference code phase $P_{r0}$.

### 3.2. Detection by Comparison

**Figure 10:**
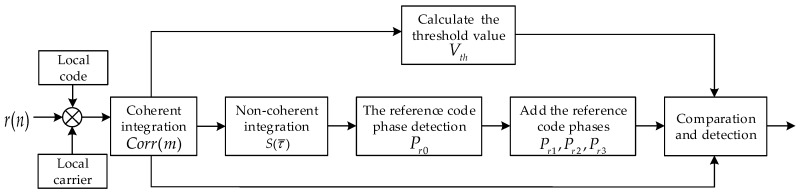
Flowchart of the MFADD.

The reference code phase can be selected by the non-coherent integration operation in the first step. The reference code phase is either previously or now aligned with the local code. If the reference phase is the start of the design signal, it can be aligned with the local code and appear *K* consecutive autocorrelation peaks with the same spacing. In hardware calculation, the autocorrelation peaks are judged by comparing the peak value with the predefined threshold. In the second step, we calculate the predefined threshold and determine the start phase of the TPRN on a more continuous comparison. In order to increase the speed of search, add another three reference code phases to search for the TPRN sequence:

(15) $$\{\begin{array}{c} P_{r1}=P_{r0}+N_{TPRN}\\ P_{r2}=P_{r0}+2N_{TPRN}\\ P_{r3}=P_{r0}+3N_{TPRN} \end{array},$$

where $P_{r0},P_{r1},P_{r2},P_{r3}$ are four reference code phases. Adaptive threshold decision method is adopted to set the set threshold value. Take the first $2N_{th}$ values of the 2*K* + 1 th correlation results, do the average filtering of $N_{th}$ points twice, multiply the minimum mean value by the coefficient as the predefined threshold:

(16) $$V_{a}=\frac{1}{N_{th}}\sum_{m=0}^{N_{th}-1}{\left|Corr(m)\right|}^{2},$$

(17) $$V_{b}=\frac{1}{N_{th}}\sum_{m=N_{th}}^{2N_{th}-1}{\left|Corr(m)\right|}^{2},$$

(18) $$V_{th}=\{\begin{array}{c} \alpha V_{a}\\ \alpha V_{b} \end{array}\begin{array}{c} V_{a}\leq V_{b}\\ others \end{array},$$

where $V_{a}$ and $V_{b}$ are the output of the average filter; α is the coefficient. The coherent integration values at these four reference code phases are compared with the predefined threshold. If the value is greater than the threshold, the counter corresponding to the phase is incremented by one, otherwise it is zeroed. The integration values make a comparison with the predefined threshold every $N_{PRN}$ chips. When one of the counter values is *K*, the next incoming chip is the start phase of TPRN, and the acquisition is successful and completed. The whole procedure is shown in Figure 10.

## 4. Performance Assessment

Based on the previous discussion, the MFADD is obtained. In this section, simulations and real data tests are performed to verify the feasibility and performance of the proposed algorithm. To achieve a comprehensive assessment of the designed signal and the proposed acquisition algorithm, Monte Carlo simulations are conducted to compare the designed signal with the conventional TPRN and verify the reliability of the algorithm. Furthermore, the designed signals are broadcasted by the MDBBS, a positioning receiver and other auxiliary equipment are also used to implement the proposed algorithm. Finally, we select several points in a building to test the positioning accuracy for the static receiver.

### 4.1. Simulations

Monte Carlo simulations are used for comprehensive evaluation of different algorithms. All simulations are implemented by M-files in MATLAB R2015a. To prove the necessity and reliability of the designed signal, comparisons are done to study the anti-MAI ability of different TPRN. To prove the effectiveness of the MFADD, static tests are performed. The designed navigation signal adopts Gold codes and characterized by the parameters in Table 1, and the sampling frequency is 10 MHz when the acquisition is processed.

**Figure 11:**
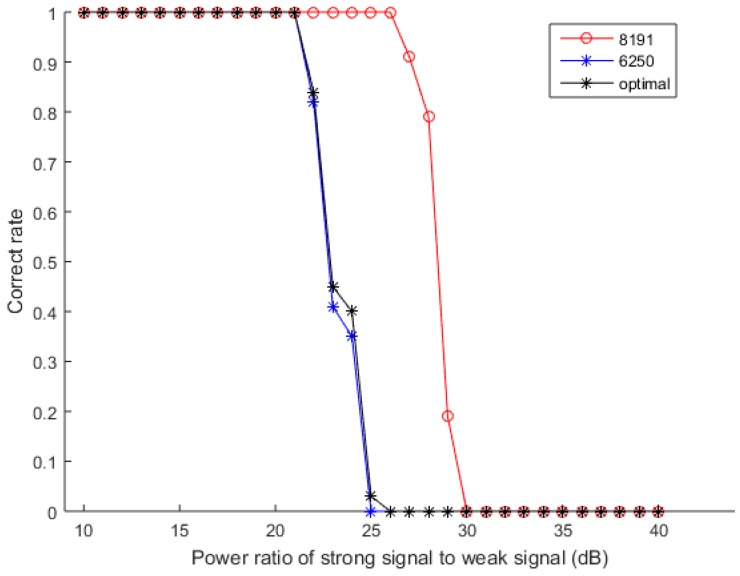
Contrast of Correct Rate of Complete Gold Code and Truncated Gold Code.

The first simulation tests the anti-MAI ability of the designed signal. To test the advantages of the designed signal, a reference signal is selected to be compared with the designed signal. Considering that the PRN length of the design signal is 8191 and the code rate is 5 MHz, the reference signal is selected 6250 chips of the same Gold code. The contrast signals are two TPRN, one TPRN truncates the complete Gold code from 8191 chips to 6250 chips by the method of Ref. [6], and the other one is the first 6250 chips of the 8191 sequence. Figure 11 illustrates the correct rate of acquiring weak signal at different signal strength with SNR = −20 dB, and the TPRN using the method of Ref. [6] is called the optimal signal. The power ratio of strong signal to weak signal is from 10 dB to 40 dB, and the simulations are repeated 100 times to obtain good statistical properties. Although the TPRN is added to the designed signal, it is actually acquired with the complete PRN, so the anti-MAI ability of the designed signal is not destroyed. Although the method used in [6] makes up for the anti-MAI performance of the optimal signal, the compensation is still limited and the anti-MAI performances of these two TPRN is close. It is found that the anti-MAI performance of the designed signal is better than that of the 5 dB reference signal in Figure 11.

**Figure 12:**
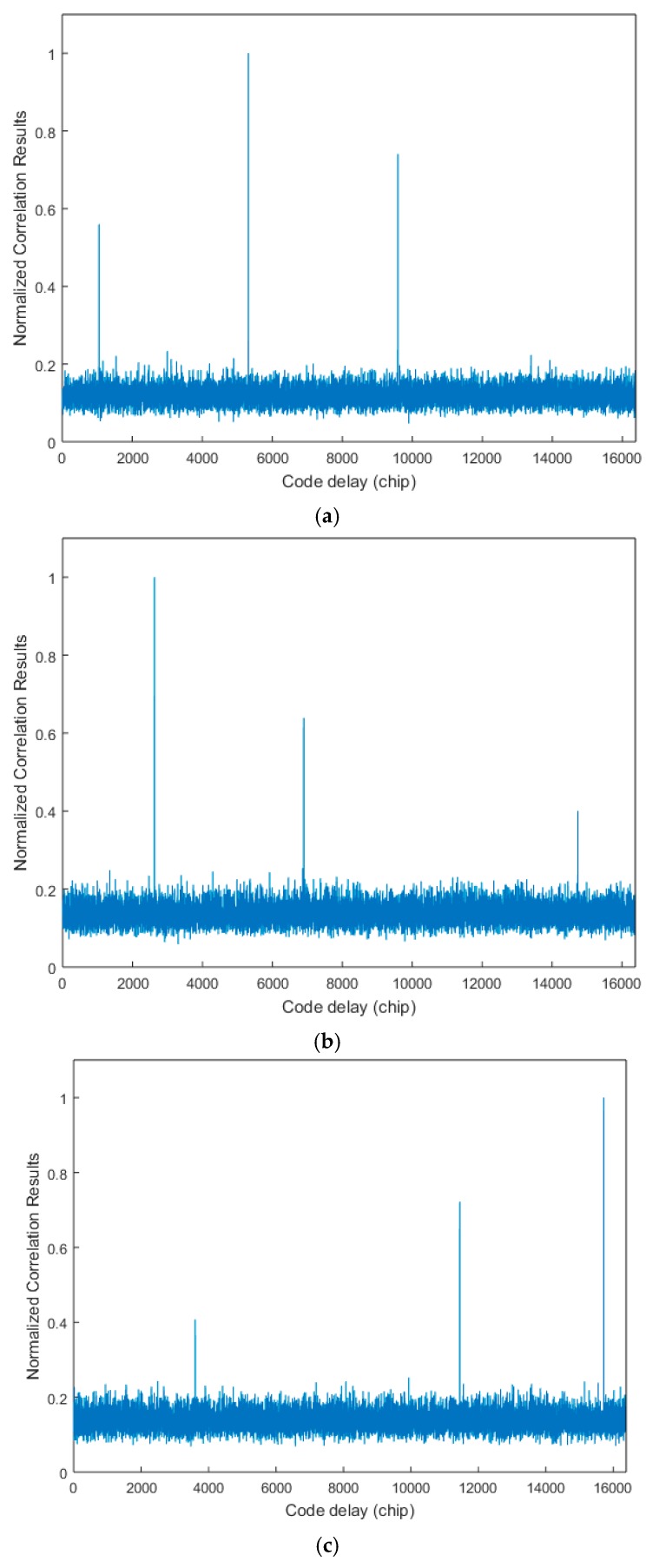

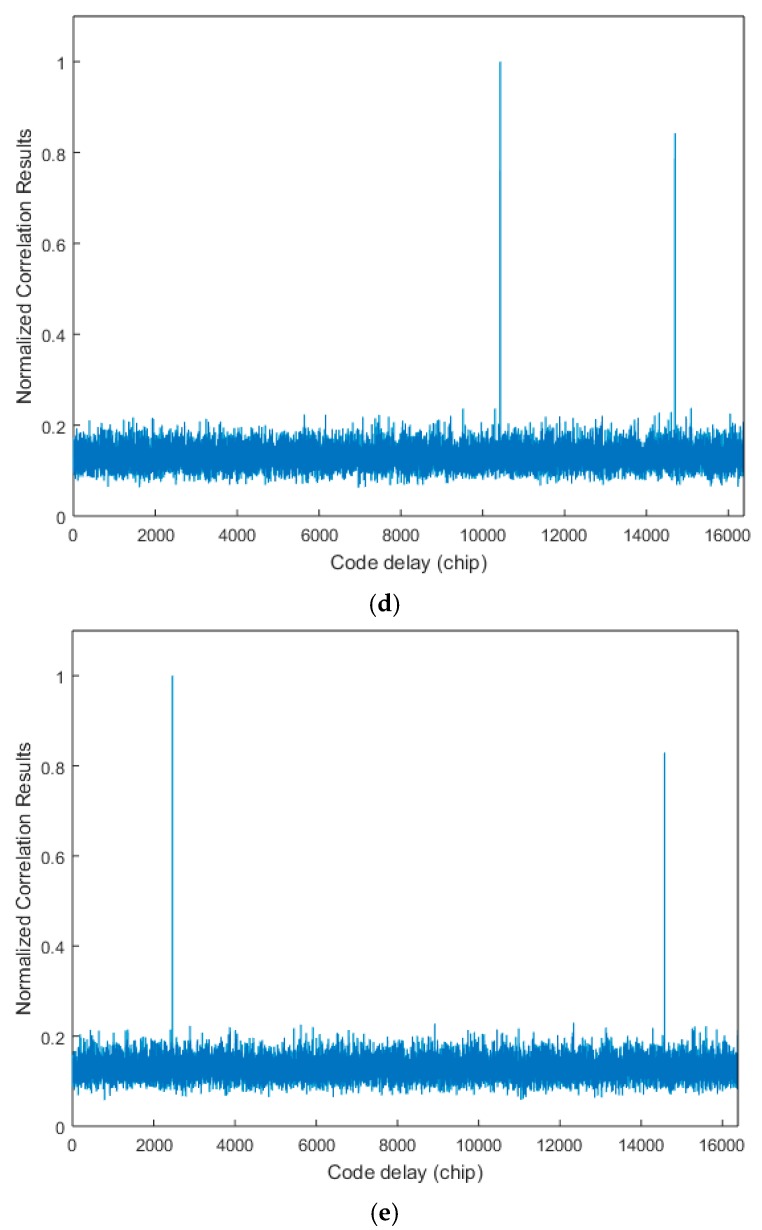
Normalized non-coherent integration of 31 non-coherent integration results of different incoming signal phase: (**a**–**c**) three significant autocorrelation peaks and (**d**,**e**) two significant autocorrelation peaks.

The second simulation verifies the abovementioned relationship of significant peak spacing. Five typical non-coherent integration results are shown in Figure 12. The incoming signal phases of these pictures are different, so the significant peak phases are different. Figure 12a–c have three significant autocorrelation peaks, peak spacing respectively satisfy Equations (9)–(11). 

In Figure 12d,e, there are two significant peaks in each picture, and the spacing respectively satisfies Equations (13) and (14). The reason for the appearance of only two significant peaks is that small autocorrelation peaks are submerged under GWN. Then the reference code phase can be detected. Adding another three code phase points by Equation (15), and continuously comparing the correlation values of these four code phases with the threshold value, the TPRN sequence phase can be detected when one of the four counter values is 15. Then the acquisition process is successfully finished. 

**Figure 13:**
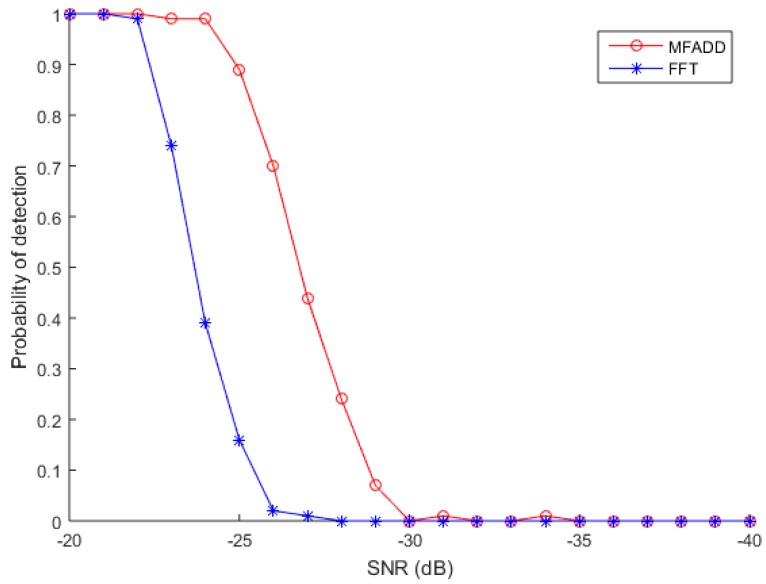
Comparison of detection probability.

The third simulation verifies the superiority of the proposed algorithm. The detection probabilities of two different schemes present in Figure 13. The comparison scheme uses the above cyclic correlation method. It can be easily concluded that MFADD has a higher anti-noise interference performance. The main reason is that there is no autocorrelation peak attenuation in MFADD, and FFT exists.

### 4.2. Real Data Tests

**Figure 14:**
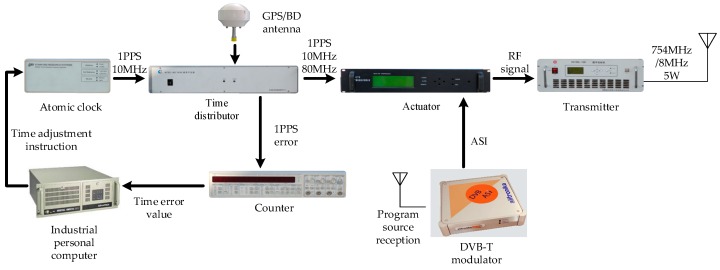
The equipment of MDBBS.

**Figure 15:**
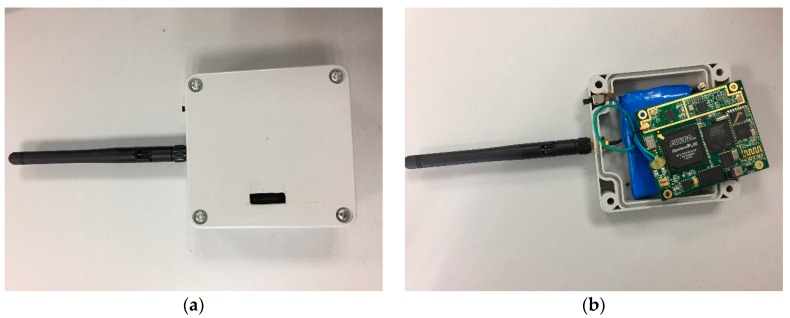
The positioning receiver: (**a**) appearance and (**b**) internal structure.

To confirm that the designed signal and MFADD exhibit better behavior, real data tests were conducted. The comprehensive test platform is shown in Figure 14 and Figure 15. The test platform consists of two parts: the MDBBS and the positioning receiver. Figure 14 details each of the pieces of equipment in the MDBBS, and the workflow of the equipment. The output frequency of the atomic clock is 10 MHz, and the time distributor, counter and industrial personal computer together control the output of the atomic clock frequency to ensure synchronization between MDBBSs. The synchronization accuracy is up to 5 ns (1δ). The time distributor contains a message generation module to generate navigation message (such as UTC time, MDBBS number, MDBBS coordinates, air pressure), and passes it to the actuator. The actuator modulates the CMMB and the navigation signal into RF signal, and finally the transmitter transmits the RF signal. Figure 15 shows the positioning receiver used for the tests, which was developed by us. The intermediate frequency signal sampler can convert the high frequency multiplexing communication and navigation signal into a zero-digital IF signal with a sampling frequency of 22 MHz. The positioning receiver uses FPGA and ARM architecture for baseband processing and demodulation, and then sends the positioning data to the host computer through the Bluetooth protocol for display. The receiver can be used to verify the effectiveness of the MFADD, and test the positioning accuracy of the entire system.

**Figure 16:**
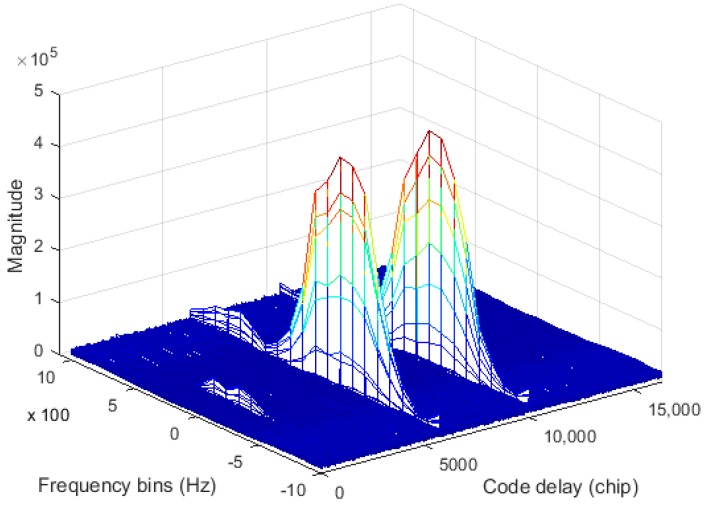
The equipment of MDBBS.

The residual carrier frequency is caused by the Doppler frequency offset and the crystal oscillator offset. The Doppler frequency offset between the receiver and the MDBBS is limited due to the fixed MDBSS and the slow motion in the indoor environment, and the crystal oscillator accuracy of the receiver depends on the temperature compensated X’tal (crystal) oscillator (TCXO). Assuming that the motion speed is 5 m/s, the carrier frequency is 754 MHz, and the TCXO accuracy is 1 ppm, the frequency offset is ${\bar{f}}_{d}\leq 767\mathrm{Hz}$. The acquisition parameters of the receiver are as follows: the frequency search range is ±1000 Hz, the frequency search step is 100 Hz, and the sampling rate is 10 MHz. Figure 16 shows the results of 31 non-coherent integration of the real data of one MDBSS. When search frequency is −100 Hz, there are three significant autocorrelation peaks on the envelope of S(⋅). Then continuously comparing the correlation values of these four code phases with the threshold value, the TPRN phase can be detected when one of the four counter values is 15.

**Figure 17:**
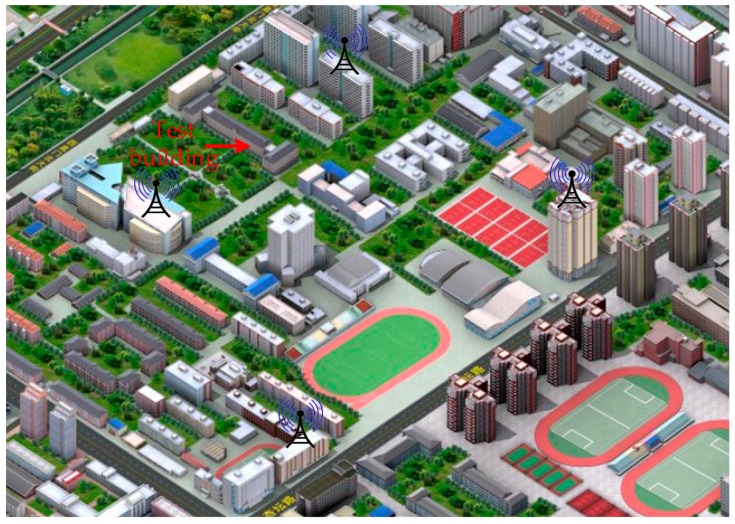
The positioning accuracy of MDBBS.

**Figure 18:**
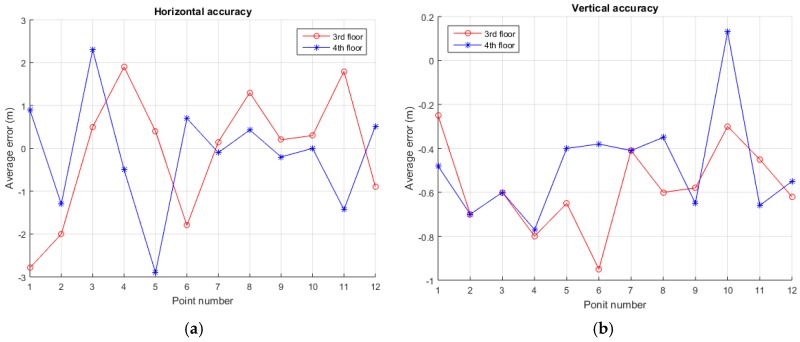
The positioning accuracy of MDBBS: (**a**) horizontal positioning results and (**b**) vertical positioning results.

In order to test the positioning accuracy of the entire system, we build an experimental environment on our campus, as shown in Figure 17. Four MDBBSs are distributed on the roof of four buildings, the 3rd and 4th floor of a teaching building are selected as the test sites. We selected 12 points on each test floor to do positioning tests, the receiver being placed at each test point for one hour tests. The horizontal positioning utilizes MFADD and the corresponding tracking algorithm, and the vertical positioning uses differential pressure measurement technology. Since the system uses a custom coordinate system for indoor positioning, the positioning results are compared with the distances of the selected points relative to the original point of the corresponding floor. The results of comparing the average value with the corresponding origin are shown in Figure 18. Figure 18a is the measurement accuracy of the distances between two points, and Figure 18b is the measurement accuracy of the vertical direction. It can be found that the distance between point and point measured is better than 3 m, and the accuracy of the vertical direction is better than 1 m. In indoor positioning, in addition to the system errors, multipath and not line of sight (NLOS) are the main influences on the positioning results. Although an adaptive filtering process has been used in the receiver, this process can only reduce the positioning errors caused by the abovementioned causes to varying degrees.

## 5. Conclusions

In this paper, a novel communication and navigation fusion signal is designed, which multiplexes the communication and navigation signals in the same frequency band. A novel algorithm named MFADD is proposed for the novel signal. Non-coherent integration of the correlation results is performed to determine the reference code phase of the presence MDBSS. Adding reference code phase points, the TPRN sequence phase is detected by continuously comparing the coherent integration values of these reference code phases with the threshold value. This algorithm has been tested by numerical simulation and real data. The test results show that the MFADD is capable of acquiring the PRN sequences with the TPRN sequence, which can improve the positioning capability of terrestrial networks and guarantee that the system’s anti-MAI ability is not destroyed. 

## Figures and Tables

**Table 1:** Simulation parameters.

Parameter	Value
slot time, $T_{F}$	25 ms
PRN sequence length, $N_{PRN}$	8191 chips
TPRN sequence length, $N_{TPRN}$	2135 chips
code rate	5 MHz
Sampling frequency, $f_{s}$	22 MHz
Intermediate frequency, $f_{IF}$	0 MHz
Residual carrier frequency, $\bar{f_{d}}$	1 kHz
Signal length	20 slot times
Data bit transition	Random
